# University of Pennsylvania

**Published:** 1850-06

**Authors:** 


UNIVERSITY OF PENNSYLVANIA.

Dr. G. B. Wood has been transferred from the chair of Materia Medica in this Institution, to that of the Theory and Practice of Medicine, vacated by the resignation of Dr. Chapman.

